# Average Nucleotide Identity and Digital DNA-DNA Hybridization Analysis Following PromethION Nanopore-Based Whole Genome Sequencing Allows for Accurate Prokaryotic Typing

**DOI:** 10.3390/diagnostics14161800

**Published:** 2024-08-17

**Authors:** Nick Versmessen, Marieke Mispelaere, Marjolein Vandekerckhove, Cedric Hermans, Jerina Boelens, Katleen Vranckx, Filip Van Nieuwerburgh, Mario Vaneechoutte, Paco Hulpiau, Piet Cools

**Affiliations:** 1 Laboratory Bacteriology Research, Department of Diagnostic Sciences, Faculty of Medicine and Health Sciences, Ghent University, 9000 Ghent, Belgium; 2 Department of Diagnostic Sciences, Faculty of Medicine and Health Sciences, Ghent University, 9000 Ghent, Belgium; 3 Department of Bio-Medical Sciences, HOWEST University of Applied Sciences, 8000 Bruges, Belgium; 4 Department of Laboratory Medicine, Ghent University Hospital, 9000 Ghent, Belgium; 5 BioMérieux, 1030 Brussels, Belgium; 6 NXTGNT, Department of Pharmaceutics, Faculty of Pharmaceutical Sciences, Ghent University, 9000 Ghent, Belgium

**Keywords:** *Escherichia coli*, whole genome sequencing, multi-locus sequence typing, antibiotic resistance, species delineation, average nucleotide identity, digital DNA-DNA hybridization, clinical bacteriology, nanopore sequencing, minimum inhibitory concentration

## Abstract

Whole-genome sequencing (WGS) is revolutionizing clinical bacteriology. However, bacterial typing remains investigated by reference techniques with inherent limitations. This stresses the need for alternative methods providing robust and accurate sequence type (ST) classification. This study optimized and evaluated a GridION nanopore sequencing protocol, adapted for the PromethION platform. Forty-eight *Escherichia coli* clinical isolates with diverse STs were sequenced to assess two alternative typing methods and resistance profiling applications. Multi-locus sequence typing (MLST) was used as the reference typing method. Genomic relatedness was assessed using Average Nucleotide Identity (ANI) and digital DNA-DNA Hybridization (DDH), and cut-offs for discriminative strain resolution were evaluated. WGS-based antibiotic resistance prediction was compared to reference Minimum Inhibitory Concentration (MIC) assays. We found ANI and DDH cut-offs of 99.3% and 94.1%, respectively, which correlated well with MLST classifications and demonstrated potentially higher discriminative resolution than MLST. WGS-based antibiotic resistance prediction showed categorical agreements of ≥ 93% with MIC assays for amoxicillin, ceftazidime, amikacin, tobramycin, and trimethoprim-sulfamethoxazole. Performance was suboptimal (68.8–81.3%) for amoxicillin-clavulanic acid, cefepime, aztreonam, and ciprofloxacin. A minimal sequencing coverage of 12× was required to maintain essential genomic features and typing accuracy. Our protocol allows the integration of PromethION technology in clinical laboratories, with ANI and DDH proving to be accurate and robust alternative typing methods, potentially offering superior resolution. WGS-based antibiotic resistance prediction holds promise for specific antibiotic classes.

## 1. Introduction

In recent years, there has been a growing interest in whole-genome sequencing (WGS) of bacterial genomes, driven by advancements in high-throughput next-generation sequencing (NGS) techniques [1,2]. WGS has emerged as a vital tool in clinical microbiology, offering comprehensive insights into bacterial pathogens. It enables typing, outbreak detection, and, significantly, the characterization of antimicrobial resistance patterns [1,3,4]. While NGS has made remarkable progress in delivering cost-efficient and timely data, the rapid evolution of this field necessitates standardization, quality control, bioinformatics tool development, and expanded utility [3,5].

Notably, the advent of third-generation NGS technologies, such as nanopore sequencing (Oxford Nanopore Technologies, Oxford, UK), marked a significant milestone. Since its release in 2014 with the MinION technology, nanopore sequencing expanded with the GridION (2017) and PromethION (2018), offering greater sequencing capacity for larger genomes [6]. While the primary objective behind the development of the PromethION technology was sequencing entire human genomes, its application in the WGS of prokaryotic genomes has, to the best of our knowledge, not yet been explored. Nevertheless, analyzing multiple prokaryotic genomes simultaneously with high confidence presents an intriguing possibility.

At present, clinical laboratories identify bacterial isolates swiftly using matrix-assisted laser desorption ionization-time of flight mass spectrometry (MALDI-TOF MS) [7]. However, this method does not provide strain (clonal) information, i.e., does not allow for typing (in a routine setting). WGS of pathogens has the potential to offer such information but remains underutilized due to its high cost, time requirements, and the need for specialized expertise and equipment [3,8]. The reference technique for typing is multi-locus sequence typing (MLST). MLST classifies a genome into a specific sequence type (ST), defined as a set of genomes sharing identical sequences (i.e., lacking single-nucleotide polymorphisms) across a small number of designated gene loci, typically 7–10 ‘housekeeping’ genes distributed throughout the genome [9,10]. Importantly, MLST has inherent limitations in that these housekeeping genes are more conserved (core genes) compared to the rest of the genome; therefore, the overall similarity of genomes in the same ST is questionable [11]. To increase the resolution, variations to MLST were introduced including whole genome MLST (wgMLST) and core genome MLST (cgMLST). The difference between these two is that wgMLST captures the entire genome sequence of an organism, encompassing both core and accessory genes, while cgMLST focuses solely on a defined set of core genes shared among closely related strains. Two other techniques that assess genomic relatedness are average nucleotide identity (ANI) and digital DNA-DNA hybridization (DDH) [11,12]. ANI and DDH are typically used to delineate species, using cut-offs of 95% and 70%, respectively. However, the extent to which these techniques can be applied for typing, in comparison with MLST, generally remains unclear.

Currently, antibiotic resistance is mostly assessed using techniques like minimum inhibitory concentration (MIC) test assays or the Kirby-Bauer test (commonly known as the disc diffusion test) [13], which both require culturing of the pathogen and take at least one to two days to perform, such that the bacterial infection is initially treated empirically. If the antibiotic susceptibility results become available, the antibiotic therapy will be adjusted if needed. In clinical microbiology, WGS has the potential to deliver results sooner than conventional phenotypic methods for antibiotic resistance prediction and could provide even more detailed data for a much bigger variety of antibiotics, especially as WGS becomes more cost-effective and data analysis becomes increasingly advanced and automated [4,5,14].

Here, we aimed to explore the potential of the PromethION sequencing technology as a tool to type bacterial strains and predict antibiotic resistance using an in-house developed and optimized wet-lab and bioinformatic workflow.

## 2. Materials and Methods

### 2.1. Bacterial Isolates

A selection of 50 bacterial isolates was made, including 48 *Escherichia coli*, one *Pseudescherichia vulneris*, and one *Klebsiella pneumoniae*. All isolates were selected from the culture collection of the Laboratory Bacteriology Research (LBR; Department of Diagnostic Sciences, Faculty of Medicine and Health Sciences, Ghent University, Belgium) and were either clinical isolates or engineered laboratory strains (expression host *E. coli*). Based on the clinical background, a strategic selection was made including both of the very closely related isolates and unrelated isolates.

### 2.2. Culture Conditions

Clinical isolates were cultured for 24 h on tryptic soy agar plates +5% sheep blood (Becton Dickinson, Erembodegem, Belgium) under aerobic conditions at 37 °C. Laboratory strains were cultured on in-house-prepared Luria–Bertani (LB) agar plates supplemented with chloramphenicol (CHL; VWR Chemicals, Radnor, PA, USA) (30 µg/mL) or ampicillin (AMP; Sigma-Aldrich, St. Louis, MO, USA) (100 µg/mL). Identification of the isolates was confirmed using MALDI-TOF MS as previously described [15].

### 2.3. Antibiotic Susceptibility Testing

Antibiotic susceptibility testing was performed on all isolates using minimum inhibitory concentration (MIC) assays (Sensititre™ Custom Plates, Thermo Fisher Scientific, Waltham, MA, USA). MIC was performed for amoxicillin (1–16 µg/mL), aztreonam (0.5–32 µg/mL), cefepime (0.5–16 µg/mL), ceftazidime (0.5–16 µg/mL), colistin (1–16 µg/mL), meropenem (1–16 µg/mL), amoxicillin-clavulanic acid (1/2–32/2 µg/mL), piperacillin/tazobactam (2/4–32/4 µg/mL), amikacin (4–32 µg/mL), tobramycin (1–16 µg/mL), tigecycline (0.25–8 µg/mL), trimethoprim-sulfamethoxazole (1/19–16/304 µg/mL), and ciprofloxacin (0.06–8 µg/mL) by using the ISO 20776-1 [16] standard broth microdilution method as recommended by The European Committee on Antimicrobial Susceptibility Testing (EUCAST) [17]. We used Sensititre™ Custom Plates according to EUCAST guidelines. We used the EUCAST 2024 clinical breakpoints to classify isolates as susceptible, intermediate, and resistant (https://www.eucast.org/clinical_breakpoints, accessed on 16 August 2024).

### 2.4. DNA Extraction

DNA extracts were prepared from all cultured isolates using the High Pure PCR Template Preparation Kit (Roche Applied Science, Basel, Switzerland). For each isolate, cells were collected from the culture plates using a sterile 10 µL inoculation loop until the eye of the loop was filled or until there were no more colonies on the culture plate to be collected. They were then suspended in a 1.7 mL Eppendorf tube containing 200 µL PBS (Lonza, Basel, Switzerland). DNA extraction was further performed according to the manufacturer’s instructions (and making use of products included in the kit) with few changes to the initial steps. Instead of using the recommended 5 µL lysozyme for lysis of the suspended bacteria followed by a 15-minute incubation step at 37 °C, 200 µL tissue lysis buffer and 40 µL proteinase K (included in the DNA extraction kit, concentration not disclosed by manufacturer) were added to each PBS suspension, briefly vortexed, and stationary incubated for 1 h at 55 °C. After this incubation, 200 µL binding buffer was added to each suspension and mixed immediately, followed by 10 min incubation at 70 °C. Further steps were performed according to the manufacturer’s protocol. The DNA extracts were stored at −80 °C.

### 2.5. Assessment of DNA Fragmentation and Concentration

The fragmentation of DNA extracts was evaluated utilizing the Fragment Analyzer^TM^ System (Advanced Analytical Technologies GmbH, Heidelberg, Germany). Visual assessment of DNA fragmentation was conducted by examining the gel view generated through fragment analysis. The mean DNA fragmentation value was selected, excluding any outliers observed in the gel, and subsequently employed for the determination of molar quantities within the whole genome sequencing protocol.

DNA concentrations were determined using two distinct instruments: the DeNovix DS–11+ Series Spectrophotometer/Fluorometer (DeNovix Inc., Wilmington, DE, USA), referred to as “DeNovix”, and a Qubit^®^ 4.0 Fluorometer (Thermo Fisher Scientific, Waltham, MA, USA), referred to as “Qubit”. The DeNovix platform was used through microvolume quantitation spectrophotometry characterized by a dynamic range of 0.75–37,500 ng/µL (dsDNA). For Qubit, a Qubit™ broad range dsDNA quantification assay kit (Thermo Fisher Scientific) was used with a dynamic range of 0.2–2,000 ng/µL (dsDNA).

### 2.6. WGS with Oxford Nanopore Technologies PromethION

DNA extracts were used for constructing a sequencing library, employing an adapted and optimized protocol. The final optimized lab protocol is provided in SI 1 in Supplementary Materials. This library was prepared using the ligation sequencing kit SQK-LSK109 (Oxford Nanopore Technologies (ONT), Oxford, UK) and the native barcoding expansion kit 96 EXP-NBD196 (ONT). A small volume of the prepared library was used for quality assessment through an initial sequencing test run on the Flongle sequencing platform (ONT). After passing this quality control by completing a successful run (data not included), the library was sequenced on the nanopore PromethION sequencing platform (ONT). A PromethION FLO-PRO002 R9.4.1 flow cell (ONT) was prepared according to the manufacturer’s guidelines using the flow cell priming kit EXP-FLP002 (ONT). The sequencing run was initiated with 8,566 available flow cell pores at the start of the run. The run length was set for 72 h, during which the flow cell was refueled with flush buffer (included in the flow cell priming) after approximately 48 h to mitigate the diminishing translocation speed. After completion of the sequencing run, the flow cell was reloaded with the same library for an additional 72 h to enhance data output in the explorative investigation function. High-accuracy basecalling was activated as a run parameter.

### 2.7. Construction of a New Bioinformatics Pipeline

Upon completion of the sequencing procedure, the raw sequence data were extracted from the PromethION platform and underwent processing through a newly developed bioinformatics Nextflow pipeline executed within a Docker environment. This pipeline was constructed by integrating publicly available tools from GitHub and in-house developed scripts. The comprehensive workflow and subsequent follow-up analyses are detailed in SI 2. The automated workflow is represented by the black square, while manual follow-up analyses are depicted outside the square.

Initial processing involved the conversion of raw sequencing data files in FAST5 format into FASTQ format using the ONT Guppy v4.0.11 basecaller, which is integrated into the PromethION sequencing platform. For each barcode, multiple FASTQ files containing WGS reads were generated and subsequently merged into a single FASTQ file per barcode. The quality assessment of the reads within the merged FASTQ files was carried out in the pipeline (see SI 2) using two tools from the NanoPack package [18]. The first tool, NanoPlot v1.33.0, is a comprehensive plotting tool designed for long-read sequencing data and alignments. It provides a statistical summary, various types of plots, and generates an HTML summary file as output [18]. The second tool, NanoComp V1.12.0, facilitates the comparison of multiple runs of long-read sequencing data and alignments [18]. These tools enable early-stage visualization of sequencing data and provide valuable insights into read length, statistical quality parameters, reference identity, and read mapping quality.

The Nextflow pipeline then utilized Flye v2.8, a de novo assembler tailored for single-molecule sequencing reads, to generate genome assembly files in FASTA format [19]. Additionally, Flye produced .txt files containing information on the number of contigs and their respective lengths. The .gfa file generated by Flye was subsequently visualized using Bandage v0.8.1, a program designed for analyzing de novo assemblies. Bandage displays connections between contigs generated by an assembler (e.g., Flye) and allows for an initial visual assessment of the assembled genome of each isolate (data not included) [20].

The assembled genomes in FASTA format created by Flye underwent a polishing step, involving correction of the data, performed by two modules integrated into the pipeline: Racon v1.4.20 and Medaka v1.2.0 (available at https://github.com/nanoporetech/medaka, accessed on 17 January 2021) [21]. These modules improved the raw contigs generated by Flye, resulting in polished consensus FASTA files containing higher-quality genomes.

The polished consensus FASTA files were subsequently employed for genome annotation using Prokka v1.14.5, which generated a .gff output file containing information on annotated genes, including those related to antibiotic resistance [22]. To validate the Prokka-based annotations of antibiotic resistance genes, they were compared to results obtained in parallel using a different online tool, ResFinder, which is further described below.

Furthermore, the merged FASTQ files underwent processing by another module, Minimap2 v2.17, to map these files against the genome assembled by Flye [23]. The output file of Minimap2 was then processed by the integrated module, Samtools v1.11, a suite of programs designed for manipulating alignment formats [24]. The .sam alignment output files from Minimap2 were converted into indexed, sorted .bam files. These .bam files were suitable for visual inspection using the Integrative Genomics Viewer v2.18.1, an interactive, user-friendly tool for exploring genomic data with high performance (data not included) [25,26,27,28].

### 2.8. Genome Quality Statistics for the WGS Assemblies

The sequencing coverage was documented from the pipeline output and was supplemented with WGS assembly quality parameters that were exported from the commercial software platform BioNumerics^®^ version 8.1 (BioMérieux, Marcy l’Etoile, France). These data were then used for the quality assessment of the WGS constructs. These quality parameters included the length of the genome, the number of contigs, the length at which half of the assembled genome is contained in contigs of that size or larger (N50), total number of ACGT bases detected, NrBAFPresent (all assembly-based calls, including perfect (100%) matches and non-perfect matches) and NrBAFPerfect (all assembly-based calls that have a perfect match with an allele in the allele database), for each isolate. We determined the ratio of NrBAFPerfect/NrBAFPresent which served as an effective measure for the quality of the sequencing data and pipeline output. These two values depend on the organism of interest and should ideally be equal to the number of known alleles for that organism [29,30]. Lower numbers could indicate significant error margins of the used techniques or data processing. Statistical analysis of these quality parameters was performed in R and visualizations were generated in Python.

### 2.9. WGS-Based Typing of Bacterial Genomes

The sequence types (STs) of the strains were determined using the open-access service tool MLST v2.0, provided by the Center for Genomic Epidemiology (CGE, Lyngby, Denmark), accessible at http://www.genomicepidemiology.org/services/, accessed on 24 August 2021. This tool applies the *Escherichia coli*#1 schema for *E. coli* utilizing 7 housekeeping genes to determine the STs and serves as the reference benchmark against which other methods will be compared [31,32,33,34,35,36,37]. Additionally, the FASTA files were also imported into BioNumerics^®^ (BioMérieux) for conducting a wgMLST allele and profile search which investigates 17,350 genetic loci based on 289 reference genomes to date to determine the STs. The MLST classifications were compared between these two techniques to investigate tool efficacy. For the final analyses, in cases where the ST could not be determined by any of the techniques, the isolates were given the closest match ST (indicated by *) or were left blank.

Two tools that are commonly used for prokaryotic species delineation, digital DNA-DNA Hybridization (DDH) [38,39], and Average Nucleotide Identity (ANI) [40], were evaluated as typing tools. The polished genomic sequences were uploaded to the DSMZ Type Strain Genome Server (TYGS) for pairwise comparison of DDH values [41]. This resulted in .csv files containing distance scores from pairwise comparisons of the uploaded genomes. For DDH analysis, the largest contigs (excluding putative plasmids) from the assemblies were submitted (in FASTA format) to the DSMZ Genome-to-Genome Distance Calculator (GGDC) server, enabling the computation of distance percentages between genomes [39,42]. Additionally, for all pairwise genomes, the ANI was assessed using the OrthoANIu tool [43]. To visualize both the DDH distances and ANI results in parallel, the Corrplot package in R was used through the MAniR tool, accessible at https://bioit.shinyapps.io/ManiR/, accessed on 4 July 2024 (source code: https://github.com/BiKC/MAniR, accessed on 4 July 2024).

### 2.10. Genomic Screening for Antibiotic Resistance Markers and Comparison with Phenotype

The FASTA files were analyzed by the CGE tool ResFinder v4.1 [32,44,45]. ResFinder was used to detect the presence of antimicrobial resistance genes against 102 distinct antimicrobials. ResFinder reports a score ranging from 0 to 3 where “0” indicates a no match found with a resistance gene in the reference database, “1” represents a resistance gene match < 100% ID and match length < reference length, “2” reports a resistance gene match = 100% ID and match length < reference length, and “3” reports a resistance gene match = 100% ID and match length = reference length. The threshold for %ID was set at 90% with a minimum length of 60% as a default. Scores ranging from 1 to 3 were all classified as a resistant genotype to the corresponding antimicrobial, or else it was classified as susceptible. Subsequently, the ResFinder predicted resistance/susceptibility phenotypes were juxtaposed with their phenotypes as assessed by MIC testing.

WGS-predicted susceptibility was compared with the phenotype as defined by MIC according to the Clinical and Laboratory Standards Institute (CLSI) guidelines [46]. Minor errors were defined as WGS-predicted susceptibility of phenotypically intermediate isolates, major errors were defined as WGS-predicted resistance of phenotypically sensitive or intermediate isolates, and very major errors were defined as WGS-predicted sensitivity of phenotypically resistant isolates.

## 3. Results

### 3.1. Characterization of the DNA Extracts

The mean DNA concentration assessed by Qubit was 362.38 ng/µL (range 93–922 ng/µL), respectively. All concentrations were within the dynamic ranges of the detectors. Fragmentation analysis of the DNA extracts in gel view is shown in SI 3. Most DNA extracts converged around the 20 kb marker with a few exceptions showing more fragmentation. Approximately 5 extracts converged around the 10 kb marker, while four DNA extracts showed more fragmentation converging around the 2 kb marker and one DNA extract around the 4.5 kb marker.

### 3.2. Protocol Optimization and Library Sequencing Features

The outcomes and discussion of the optimizations to the lab protocol are described in detail in SI 4.

### 3.3. Characterization of the WGS Data

#### 3.3.1. ANI and DDH Percentage Identities Compared to MLST

The pairwise ANI and DDH values are presented by a similarity matrix together with the STs defined by the reference typing technique MLST in SI 9. Symmetry across the diagonal indicated the ANI and DDH techniques to be very concordant. DDH sequence similarities were in the range of 69.5–100% whereas the ANI analysis exhibited a narrower range of overall similarity with a range of 95.9–100%. Importantly, the concordance between the MLST classifications and the ANI and DDH clusters was evident, as MLST tended to align with both specific ANI and DDH clusters, as marked by squares along the diagonal in the matrix. The minimal percentage identity for both ANI and DDH for all genomes classified as the same MLST are shown in Table 1, MLST represented by only a single genome were excluded from this analysis. Based on these minimal ID percentages, it is possible to suggest cut-off ANI and DDH values for intraspecies delineation of strain types. For ANI, we observed a minimal ID% of 99.32% whereas for DDH this was 94.10%.

#### 3.3.2. Determination of the Minimal Sequencing Coverage Required for Qualitative Analyses

The WGS data was substantial in size (>2 TB of raw data); therefore, we investigated possible reductions in raw data for future analyses while maintaining the results for key WGS features such as genome size, typing data, and antibiotic resistance. We aimed to identify the minimally required sequencing coverage that did not affect these WGS features compared to those from the same isolates but with higher WGS coverages.

We performed pairwise assessments of the WGS features for nine isolates between the complete dataset (containing 100% of the data) and six subdatasets derived from the complete dataset to determine the minimally required sequencing coverage. The nine isolates were selected based on their ST and genomic relatedness in the MLST–ANI–DDH similarity matrix as depicted in SI 9. The nine isolates were selected from different STs and different MLST–ANI–DDH similarity matrix clusters as defined by a minimal ID% of 99.32% (ANI) or 94.10% (DDH) (SI 9 and Table 1). The six subdatasets were defined by fixed percentages from the complete dataset from 100% to 3% in 6 steps (100%, 75%, 50%, 25%, 10%, 5%, and 3%) which could also be expressed as the percentage reduction of the absolute number of raw read data files. The six subdatasets were compiled accordingly using the subsampling feature of the seqtk tool (https://github.com/lh3/seqtk). The results of these pairwise assessments of WGS features between the complete dataset and the six subdatasets are shown in SI 6.

The length of the longest contig (i.e., a measure for genome size) remained relatively consistent with a decreasing percentage of reads down to a coverage/percentage reads of 12× and 3–5%, respectively. An empirical approach was applied to determine significant differences between the six subdatasets where the ANI/DDH value of the first subdataset—the mean of the current and previous subdatasets had to be >0.02% for ANI, and >0.1% for DDH. These cut-off values were chosen based on the overall assessment of the six subdatasets. Generally, significant differences in key features were observed for coverages <12× which translated to a subdataset containing 3–5% of raw reads.

Subsequently, we created a new dataset containing 10% of the data of the complete dataset (hereinafter referred to as the “reduced dataset”—using the same subsampling method—and containing all isolates from the complete dataset. The pipeline-processed reduced dataset was then pairwise compared to the complete dataset to investigate the concordance of features including genome length, number of contigs, number of bases, N50, the ratio of NrBAFPerfect/NrBAFPresent, typing, and antibiotic resistance detection.

#### 3.3.3. Pairwise Analysis of WGS Features between the Complete and the Reduced Datasets

**Figure 1:**
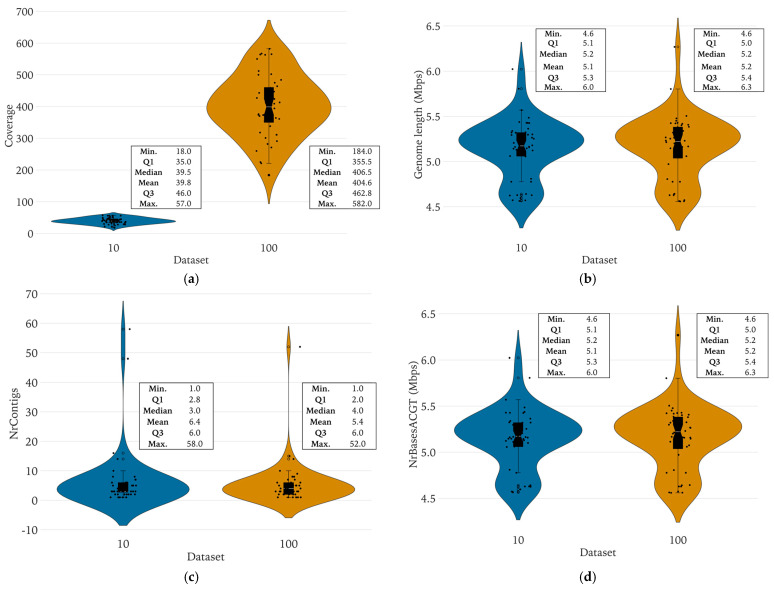

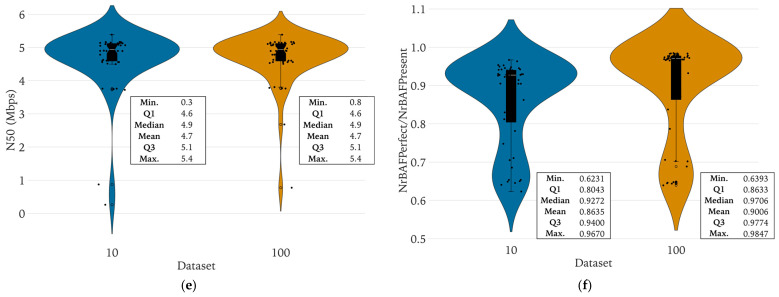
Pairwise comparison of WGS quality parameters between the complete and reduced datasets. (**a**) The sequencing coverage (×); (**b**) genomic length (Mbps); (**c**) number of contigs; (**d**) total number of nucleic acid bases; (**e**) N50 (Mbps); (**f**) NrBAFPerfect/NrBAFPresent ratio, for the complete dataset (100% of raw output data) and the reduced dataset (10% of raw output data). Each genome quality parameter is presented by a violin plot juxtaposed with a strip plot for both datasets.

Pairwise analyses of distinct WGS quality parameters are shown in Figure 1 for both the complete dataset and the reduced dataset, only encompassing the *E. coli* WGS in our study. Summary statistics are documented in SI 7 and SI 8. In summary, we found no statistically significant differences for any of the parameters, except for the coverage, evidently.

For the complete dataset, the mean sequencing coverage was 404.6× (range 184–582×), and a median coverage of 406.5×. The reduced dataset displayed a mean coverage of 39.8× (range 18–57×) with a median coverage of 39.5×. The mean genome size was 5,163,046 bps (range 4,558,973–6,268,206 bps) for the complete dataset, compared to 5,145,316 base pairs (range 4,564,212–6,022,166 bps) for the reduced dataset. The genomes were visualized with Bandage and presented either a circular shape (42 isolates) or an irregular shape (8 isolates) (data not included). The mean number of contigs in the complete dataset was 5.4 (range 1–52) with a median of 4 contigs while the reduced dataset exhibited a higher mean of 6.4 contigs (range 1–58) and a median of 3 contigs. The mean number of bases for the complete dataset was 5,163,046 bases (range 4,558,973–6,268,206 bps), while the reduced dataset exhibited a mean of 5,145,316 bases (range 4,564,212–6,022,166 bps). The mean N50 value was 4,703,756 bps (range 772,895–5,385,327 bps) and 4,651,574 bps (range 259,382–5,385,990 bps), for the complete and reduced datasets, respectively. The NrBAFPerfect/NrBAFPresent ratio exhibited a mean of 0.90 (range 0.64–0.98) for the complete dataset and a mean of 0.86 (range 0.62–0.97) for the reduced dataset.

#### 3.3.4. Pairwise Assessment of Strain Typing with MLST and ANI for the Complete and the Reduced Datasets

**Figure 2:**
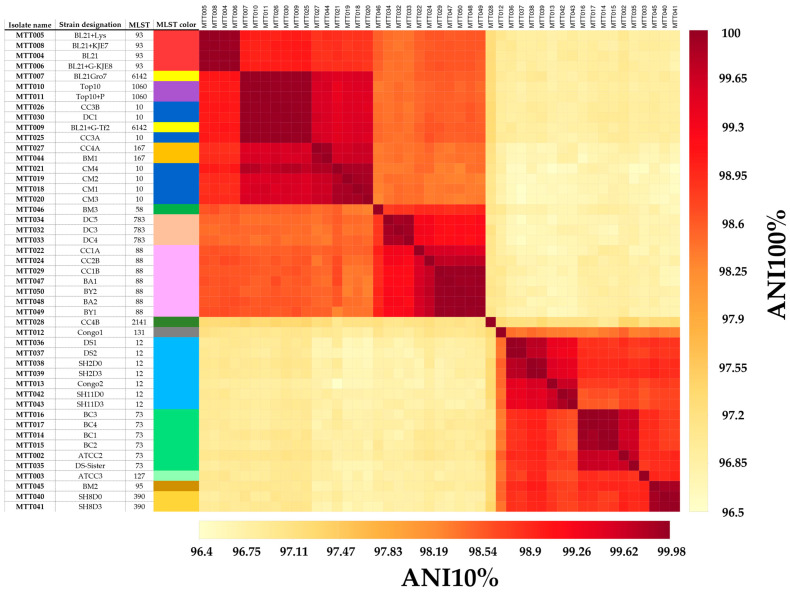
Similarity matrix depicting Average Nucleotide Identity (ANI) for the complete and reduced datasets juxtaposed with multi-locus sequence types (MLST). The matrix illustrates ANI values with the bottom left section representing the reduced dataset and the upper right section displaying the ANI data of the complete dataset. The analysis includes 47 *E. coli* isolates, after the omission of one outlier isolate (MTT023). On the left side, isolate IDs (two left columns) are presented alongside their CGE MLST (third column), with each unique MLST class color-coded for distinction. (Visualization: https://bioit.shinyapps.io/ManiR/, accessed on 4 July 2024).

The pairwise analysis of ANI values from the complete and reduced datasets is presented together with the STs (as assessed using the CGE tool) in Figure 2. Isolate MTT023 was excluded from the results due to its disproportionate influence on the reduced dataset, resulting in a distorted color scale that ranged from 65% to 100% (figure not shown). This disparity stemmed from the poor sequence quality of MTT023 and was likely caused by contamination of the sample. Nonetheless, MTT023 had a sequencing coverage of 271×/28× (100%/10% dataset) but yielded suboptimal assembly results, yielding a highly fragmented and non-circular genome comprising a total of 50 contigs. Remarkably, the primary contig was only 1,617,761 bps in length, a rarity within the complete dataset where primary contig lengths typically exceeded 4 Mbps. Given these distinctive attributes, data reduction to 10% of the original data for MTT023 would be detrimental, rendering it unsuitable for subsequent analyses.

Symmetry across the diagonal of the similarity matrix indicates the ANI values of both datasets to be highly similar. The ANI sequence similarities were in the range of 96.5–100% and 96.4–99.98% for the complete and reduced datasets, respectively. The differences in ANI similarity values between the complete and the reduced datasets had a median of 0.0103% and ranged from −0.3277% to 0.2163%. Notably, here we also observed strong concordance between the MLST classifications and the ANI clustering from both datasets.

MLST classification using the CGE and BioNumerics tools showed ST agreement in 45 out of 48 isolates (93.8%) for the complete dataset and 40 out of 48 isolates (83.3%) for the reduced dataset (SI 10). In 2 (4.2%) and 3 (6.3%) out of 48 isolates in the complete and reduced datasets, respectively, the ST could not be identified by any of the tools. These 2 and 3 cases, respectively, were different isolates between the two datasets.

In the complete dataset, for 2 out of 48 isolates (4.2%) the ST was not identified by the CGE tool, and another 3 assigned STs were marked as questionable (6.3%). In the reduced dataset, for 3 out of 48 isolates (6.3%) the ST was not identified, and 8 out of 48 isolates (16.7%) were assigned questionable STs. BioNumerics was not able to assign an ST in 5 out of 48 isolates (10.4%) and 11 out of 48 isolates (22.9%) in the complete and reduced datasets, respectively. Unlike the CGE tool, BioNumerics did not provide the closest ST match.

#### 3.3.5. Pairwise Analysis of Antibiotic Resistance Genes in the Complete and Reduced Datasets

The pairwise comparison of WGS-based predictions of antibiotic resistance with the resistance phenotypes for 13 antibiotics for all 48 *E. coli* isolates between the complete and reduced datasets is summarized in Table 2 with visual representation in SI 11. For the complete dataset, 76 out of 624 (12.18%) were wrongly predicted (i.e., minor, major, or very major errors) by WGS. Of these 76 discrepancies, 3 (0.48%) were classified as minor errors (MIC/WGS: I/S), 19 (3.04%) as major errors (MIC/WGS: S/R and I/R), and 54 (8.65%) as very major errors (MIC/WGS: R/S). In the reduced dataset, 73 discrepancies were observed (11.70%). Of these, 4 (0.64%) were classified as minor errors, 19 (3.04%) as major errors, and 50 (8.01%) as very major errors. A more detailed examination per isolate is shown in SI 11.

The accuracy of the WGS-based prediction of antibiotic resistance versus the reference MIC assay in the complete dataset is antibiotic-dependent (Table 2). High accuracies above 90% were observed for all the antibiotics in the classes carbapenems, aminoglycosides, tetracyclines, and miscellaneous agents such as colistin and trimethoprim-sulfamethoxazole. Very high accuracies were observed for the following antibiotics: amoxicillin (97.8%), ceftazidime (95.8%), meropenem (100%), amikacin (95.8%), and trimethoprim-sulfamethoxazole (97.9%). Antibiotics where accuracy was lowest included amoxicillin-clavulanic acid (68.8%), piperacillin-tazobactam (75.0%), cefepime (81.3%), aztreonam (68.8%), and ciprofloxacin (79.2%).

Both datasets were largely concordant with only 12 out of 624 (1.92%) discordant results. Notably, 6 out of 12 discrepancies were registered to isolate MTT028 (SI 11). The sequencing data for MTT028 revealed significantly inferior sequence quality, characterized by genome files containing 15 and 16 contigs in the complete and reduced datasets, respectively. Concordance between the datasets was also the case for the prediction accuracy.

## 4. Discussion

In this study, we investigated the potential of WGS using the PromethION nanopore sequencing platform for the typing of bacterial isolates and the determination of antibiotic resistance profiles. Our research aimed to contribute to the development of streamlined protocols that could facilitate the integration of WGS into (clinical) routine diagnostic workflows thereby enhancing our ability to monitor and intervene in nosocomial outbreaks and to understand antibiotic resistance.

### 4.1. An Optimized Protocol for the PromethION Whole-Genome Sequencing of Prokaryotes

Our study represents an important contribution to the field of microbial genomics by optimizing the PromethION nanopore sequencing technique for the sequencing of prokaryotic genomes. While this approach involves adapting an existing GridION protocol for a new platform, it provides valuable insights and practical applications for researchers utilizing the PromethION system. To the best of our knowledge, this specific application has not been previously documented. We encountered several challenges during the preparation of the library, primarily related to low DNA recovery after each step in the protocol, making it imperative that we optimized the manufacturer’s recommended lab protocol for the MinION/GridION library preparation (up to 96 barcodes) for the PromethION library preparation. Through protocol adjustments, we successfully adapted the PromethION method to suit prokaryotic whole genome sequencing. These optimizations primarily entailed (i) commencing with a fivefold DNA quantity, (ii) extended incubation times (iii) an adjustment in the DNA-binding beads to pool volume ratio to 1:1 instead of 0.4:1, and (iv) an increased elution volume in the last step of the protocol. We found a notable 32.4% increase in DNA recovery which is likely an underestimation given that we began with twice the amount of DNA from the initial protocol before implementing these optimizations.

The increased start quantity of DNA in combination with extended incubation times and an adjusted bead-to-pool ratio led to a significantly improved reaction efficiency of 56.6% and 13.9% increase observed for the first and second step of the protocol, respectively. Prior studies have discussed DNA recovery performance using Beckman Coulter Agencourt AMPure XP beads (Beckman Coulter, Brea, CA, USA) after the cleanup steps, which is based on the principle of nucleic acid binding to solid-phase reversible immobilization (SPRI) beads [47,48,49,50,51]. The manufacturer’s product information for AMPure XP beads indicates that the most common bead-to-sample ratio for NGS library contaminant removal is 1.8×). Adjusting the bead-to-sample ratio allows for size selection of DNA fragments during cleanup [48]. Lower ratios (<1.0×) favor long DNA fragment selection, while higher ratios yield smaller DNA fragments [48]. Furthermore, we eluted the sample with 26 µL of elution buffer instead of the recommended 15 µL, an important optimization of the source protocol since DNA recovery depends on factors including DNA fragmentation, sample volume and concentration, and the elution volume (Beckman Coulter, 2013).

Finally, we loaded the flow cell with 7.4 µL of the library corresponding to 588 ng DNA or 50.29 fmol DNA, as per the manufacturer’s recommendation to load the PromethION flow cell with 5–50 fmol or 300 ng of DNA, and repeated this after 72 h to maximize data output for explorative data analysis. Instead of using absolute DNA weight, we based our approach on molar quantities (calculated using a mean DNA fragment length of 20 kb based on the DNA fragmentation assay), based on our experience with nanopore sequencing. The goal was to maximize the DNA pool for each isolate to 50 fmol thus corresponding to 1 fmol/isolate in equimolar quantities.

### 4.2. ANI and DDH Are Robust Strain Typing Methods for E. coli

Typing is vitally important for epidemiologic surveillance and timely intervention for the implementation of targeted control measures during outbreaks of pathogenic bacteria. The reference technique for typing, MLST, and its variants (including wgMLST and cgMLST) are inherently flawed and exhibit differences in discriminatory resolution and utility [11]. Nonetheless, previous studies indicate that these different approaches often demonstrate similar results, making it unclear which technique is superior [8,52,53,54]. This stresses the need for alternative methods to delineate strains within a species. Therefore, we investigated if ANI and DDH, two essential techniques to investigate relatedness between genome sequences, can be used for strain typing. The advantages of these two methods are that they do not require fully assembled genomes to determine the ANI–DDH values and always provide a similarity percentage as a result, in contrast to MLST where there is a need for MLST schemes to determine STs (which are limited) and where STs are also often missing.

Firstly, we observed sequence similarity ranges of [95.9–100%] for ANI, and [69.5–100%] for DDH, which is in line with previous studies stating a minimum of 95% and 70% for species delineation using ANI and DDH, respectively.

Notably, we found that (i) the ANI and DDH technique are comparable to discriminate strains, and that (ii) we could successfully discriminate the WGS of our isolates into different ANI–DDH clusters (or groups). The results of the MLST reference technique (i.e., the STs of the isolates) superposed on the ANI–DDH results indicated that the ANI–DDH clusters align with certain ST classifications, therefore, MLST could be used to annotate these clusters as distinctive identities. Cut-off values could be defined for intraspecies delineation based on the ANI–DDH clusters with an ANI cut-off of 99.3% sequence similarity, whereas for DDH this was 94.1%.

Importantly, we also found that for ANI and DDH the discriminative resolution was potentially better than the MLST reference technique. Within certain STs (ANI ≥ 99.3% or DDH ≥ 94.1%), we were able to discriminate strains even further. Further investigation of technical replicates and how they are located within a certain cluster might reveal more intricate details regarding this potentially better resolution. Notably, since strains within the same ST tend to cohere to a specific ANI or DDH cluster if the ST could not be identified for one or more strains, strain discrimination might still be feasible based on the ANI-DDH cluster to which the isolate belongs. These findings indicate that at least for *E. coli*, ANI and DDH are potent and robust strain typing methods with a potentially better discriminative resolution, and parallel use of the MLST reference technique can be considered an interesting method to annotate the ANI–DDH data into more reader-digestible results.

In addition to ANI and DDH, SNP assays can also be considered for strain discrimination which can provide high-resolution insights into genetic variations, especially related to antimicrobial resistance. The high resolution of an SNP-based approach derives from the inclusion of intergenic regions and without compressing multiple genetic changes into a single allele, in contrast to traditional MLST approaches [55]. However, both SNP assays and traditional MLST methods require reference genomes or schemes to which the raw data can be mapped, in contrast to ANI–DDH analysis which does not require such references as they compare entire genome sequences directly to calculate similarity percentages [5,55,56,57]. Recent advancements, such as PromethION technology, offer promising alternatives by allowing high-throughput sequencing with relatively lower costs and quicker turnaround times. Integrating WGS with methods like ANI, DDH, and MLST could enhance strain typing accuracy and efficiency, providing a more comprehensive understanding of microbial populations and their resistance profiles. Regarding cost-effectiveness, PromethION’s ability to reduce sequencing costs by scaling up the number of genomes that can be sequenced while maintaining high accuracy makes it a valuable tool in both high-tech and resource-limited settings, potentially democratizing access to advanced microbial typing techniques.

### 4.3. WGS-Based Prediction of Antibiotic Resistance Compared to MIC Assay Predicts with >90% Accuracy for Some Classes of Antibiotics

To investigate the usefulness of PromethION-derived WGS to acquire clinically important information, such as antibiotic resistance, we used the WGS to predict the antibiotic resistance phenotype and compared this with the results from the reference MIC assay.

Our findings indicate a high categorical agreement for certain antibiotics. More specifically, we found a categorical agreement of ≥93% for amoxicillin (97.9%, based on 15 sensitive (S) and 32 resistant (R) isolates), ceftazidime (95.8%, 28S, 18R), amikacin (95.8%, 43S, 3R), tobramycin (93.8%, 35S, 10R), and trimethoprim-sulfamethoxazole (97.9%, 34S, 13R). For the antibiotics meropenem (48S), tigecycline (45S), colistin (45S), and piperacillin-tazobactam (36S), our dataset lacked phenotypically resistant isolates making it impossible to assess the performance of genotypical prediction of phenotypical resistance. The antibiotics amoxicillin-clavulanic acid (22S, 11R), cefepime (38S, 1R), aztreonam (26S, 7R), and ciprofloxacin (31S, 7R) showed significantly lower categorical agreements (68.8–81.3%).

Our findings are in line with previous studies, comparing phenotypic assay results with WGS results by the ResFinder tool [44,58,59]. These reported mean categorical agreements of ≥94% for *E. coli* isolates and this varied depending on the antibiotics tested, testing conditions, and profile selection of the isolates [44,58,59]. Vanstokstraeten et al. reported high categorical agreements (>95%) for the antibiotics amoxicillin, cefepime, cefotaxime, ceftazidime, amikacin, and tobramycin, and low categorical agreements (<95%) for the antibiotics amoxicillin/clavulanic acid, piperacillin/tazobactam, and ciprofloxacin, which is in line with our findings for amoxicillin, ceftazidime, amikacin, tobramycin, amoxicillin-clavulanic acid, and ciprofloxacin [58]. For cefepime, we found discordant results, however, we only had one phenotypically resistant isolate which is most likely not sufficient to accurately assess the categorical agreement. Previous studies also reported difficulties in testing and interpretation of antibiotics like amoxicillin-clavulanic acid and piperacillin-tazobactam due to a high area of technical uncertainty when using the EUCAST breakpoints [60]. An explanation for the low sensitivity of ciprofloxacin might be related to its antibiotic resistance mechanism which is very complex and difficult to predict solely based on individual resistance genes. Resistance based on point mutations might not be picked up by the sequencing method or during data analysis [61]. Importantly, in the case of the identified errors, it is likely that some of the errors might emanate from the resistance classification by ResFinder, which hinges on the match percentage of sequence similarity and the length match between the gene of the isolate and the reference resistance gene in the database. Resistance classifications with 1, 2, and 3, all culminate in a resistant profile. However, for cases classified as 1 or 2, the resistance gene sequence was only partially detected in the tested WGS, potentially indicating mutations or deletions in the resistance gene that could make the gene ineffective. A previous study highlighted discrepancies between phenotype observations and WGS predictions due to improper classification or misidentification of resistance genes, alongside limitations in the reference database of ResFinder [62].

### 4.4. A Minimal Sequencing Coverage of 12× Was Required to Maintain Key Features

The massive quantity of raw sequencing data posed challenges in data transfer and analysis, especially when working towards a protocol implementable in a clinical laboratory. Therefore, we investigated the quality of the data in relation to data quantity. Here, we focused on the minimal sequencing coverage that maintains comparable qualitative results including genomic key features (i.e., genome length) and typing (i.e., ANI and DDH) results.

We found that a sequencing coverage ≥12×, which roughly translates to 3–5% of the original dataset of the analyzed strains in this study (*N* = 9), was sufficient to maintain the length of the longest contig, ANI, and DDH values. Coverages <12× either impacted the length of the longest contig or the ANI and DDH values. Interestingly, this is identical to the results from a previous study which describes a minimum sequencing coverage of 12× required to accurately call the STs with lower coverages resulting in much more variations [63].

Consequently, as a follow-up analysis, we composed a new ‘reduced’ dataset comprising 10% of the raw read data, ensuring some tolerance on variability between all of the strains and with a mean coverage of ± 40×, for a more expanded and detailed investigation in genome quality statistics, typing, and antibiotic resistance results.

### 4.5. There Were No Significant Differences in Genomic Key Features, Typing, and Antibiotic Resistance Prediction between the Complete and Reduced Datasets

In the pairwise analysis between the complete and reduced datasets, we found a mean coverage of 404.6× and 39.8×, respectively. Other features including genome size, number of contigs, number of sequenced bases, N50, and the NrBAFPerfect/NrBAFPresent ratio all exhibited concordant results across both datasets and were not significantly affected by data reduction. Genome sizes presented means of 5.16 and 5.15 Mbps in the complete and reduced datasets, respectively. This aligns with the *E. coli* reference genome length of 4.5–5.5 Mb as documented in the literature [64]. Importantly, the NrBAFPresent parameter, an indicator for the number of alleles recognized, indicated a mean of 4199 genes for both datasets. This is also in line with previous research reporting a total estimated *E. coli* gene count between 4000–4400 genes [29,30].

There was no significant impact observed on typing results between both datasets. This is as expected since the reduced dataset had a mean coverage of 39.8× and is still fourfold the minimally required coverage of 12× to accurately call STs that we found during our data reduction strategy. Notably, few differences in MLST classification were observed between the two datasets; however, they were minimal and were not necessarily caused by data reduction since classification did not show superiority for the complete dataset. Interestingly, the ANI and DDH typing results were unaffected, underscoring the robustness of the ANI/DDH methods for typing.

Finally, no significant differences were observed between the complete and reduced datasets in terms of WGS-based antibiotic resistance prediction compared to the reference MIC assay. Between the two datasets, 1.92% of the observations were discrepant of which half are attributed to isolate MTT028. The sequencing data for MTT028 revealed significantly inferior sequence quality, characterized by genome files containing 15 and 16 contigs which is significantly higher than the median of 4 and 3 for the 100% and 10% datasets (both outliers), respectively, which might explain these discrepancies.

In summary, we found that a mean coverage of 40× was sufficient to perform our typing and antibiotic resistance analyses and further reduction of this coverage should also present similar qualitative results as long as the coverage is ≥12×. For future perspectives, this would imply that (i) the sequencing time of WGS can be further reduced to a minimum if the minimally required coverage is reached, and (ii) lower raw data volumes are needed.

### 4.6. The Choice of MLST Classification Tool Determines the Robustness of the Results

This study was not designed to compare MLST classifications by different tools, but we found that the CGE and BioNumerics tools largely agreed in assigning STs in the complete dataset. The CGE tool could allocate an ST to most isolates, mainly because of its ability to provide the closest match percentage ST, which in most cases was also the true ST (when analyzing the reduced dataset and based on STs from the complete dataset, and/or suggested by ANI–DDH clusters). This demonstrates that the CGE tool is a more robust classification tool compared to BioNumerics.

Challenges of these tools include the up-to-date status of the reference databases, computing efficiency, false positive results, difficulties in calling alleles in samples with low sequencing coverages, and variable performance with mixed samples [63]. Generally, these tools were shown to perform moderately well with low mean genome coverage and, in some cases, might also report low confidence with contaminated samples [63]. The few differences we observed are highly likely related to the above challenges some of these tools face, such as the ability of the CGE tool to provide the closest MLST matches, in contrast to BioNumerics [63]. It is also important to note that in cases where both tools could not assign an ST, it could also be due to the presence of genomes representing undefined STs, which is not a limitation of the methods but an indication of novel or uncharacterized strains.

Lastly, we did not observe significant differences between the tools in ST classification solely based on their underlying method of determining the ST, with the CGE tool focusing on only 7 housekeeping, whereas BioNumerics performed a wgMLST analysis, including many core and accessory genes. This is in line with previous studies which demonstrated that the results from MLST, wgMLST, and cgMLST are often quite similar [8,52,53,54].

## 5. Conclusions

This study demonstrates the successful adaptation and optimization of a GridION library preparation protocol for the PromethION nanopore sequencing platform for whole-genome sequencing (WGS) of bacterial isolates. The use of Average Nucleotide Identity (ANI) and DNA-DNA Hybridization (DDH) for strain typing showed equal (and possibly superior) discriminatory resolution compared to MLST techniques. Additionally, WGS-based predictions of antibiotic resistance achieved high accuracy for several antibiotic classes compared to phenotypic assays. Importantly, a minimum sequencing coverage of 12x was identified as sufficient for maintaining key genomic features and accurate typing results, indicating that data reduction strategies can be effectively implemented without compromising data quality. The choice of the MLST classification tool also influenced the robustness of the results, with the CGE tool outperforming BioNumerics.

### Study Limitations

We only included a rather limited number of *E. coli* isolates, which did not represent all of the *E. coli* diversity, and the extent to which our findings can be generalized to a broader range of STs needs further study.

## Figures and Tables

**Table 1:** ANI and DDH thresholds for MLST classification. ANI, average nucleotide identity; DDH, digital DNA-DNA hybridization; (ML)ST, (multi-locus) sequence type.

MLST	N_genomes_	%ANI_min_	%DDH_min_	Remark
ST93	4	99.94	100	Aligns with a single ANI/DDH cluster
ST6142	2	99.95	99.90	Aligns with an ANI/DDH cluster also containing ST1060, ST10
ST1060	2	99.98	100	Aligns with an ANI/DDH cluster also containing ST6142 and ST10.
ST10	6	99.87	98.9	Aligns with two ANI/DDH clusters with one cluster containing only ST10 and one cluster containing ST6142 and ST1060.
ST167	2	99.91	99.70	Aligns with a single ANI/DDH cluster and is closely related to ANI/DDH clusters containing ST6142, ST1060, and ST10.
ST88	8	99.36	95.00	Aligns with a single ANI/DDH cluster. Further discrimination is possible based on ANI/DDH.
ST783	3	99.90	99.60	Aligns with a single ANI/DDH cluster.
ST12	7	99.32	94.10	Aligns with a single ANI/DDH cluster. Further discrimination is possible based on ANI/DDH.
ST73	6	99.62	96.70	Aligns with a single ANI/DDH cluster. Further discrimination is possible based on ANI/DDH.
ST390	2	99.96	100	Aligns with an ANI/DDH cluster also containing ST95 (*N* = 1).

**Table 2:** Summary of the results of the pairwise comparison of phenotypically observed and WGS-predicted antibiotic resistance between the complete (100%) and reduced (10%) datasets. Tested antibiotics are shown on the left side of the table (ordered by EUCAST nomenclature). The percentages of isolates are shown for the phenotypic MIC assay as RIS classification and for the WGS-predicted genotype antibiotic resistance/susceptibility. The categorical agreement refers to the percentage of isolates where the interpreted RIS classification conforms to the results of the phenotypic assay.

	% Susceptible Phenotype (MIC)	% Intermediate Resistant phenotype (MIC)	% Resistant Phenotype (MIC)	% Susceptible Genotype		% Resistant Genotype		% Categorical Agreement		Minor Errors (%)		Major Errors (%)		Very major Errors (%)	
				100%	10%	100%	10%	100%	10%	100%	10%	100%	10%	100%	10%
**PENICILLINS**															
Amoxicillin	31.3	0.0	68.8	33.3	31.3	66.7	68.8	97.9	100.0	0.0	0.0	0.0	0.0	2.1	0.0
Amoxicillin-Clavulanic Acid	45.8	0.0	54.2	77.1	77.1	22.9	22.9	68.8	68.8	0.0	0.0	0.0	0.0	31.3	31.3
Piperacillin-Tazobactam	75.0	0.0	25.0	100.0	97.9	0.0	2.1	75.0	77.1	0.0	0.0	0.0	0.0	25.0	22.9
**CEPHALOSPORINS**															
Cefepime	91.7	4.2	4.2	81.3	81.3	18.7	18.8	81.3	79.2	0.0	2.1	16.7	16.7	2.1	2.1
Ceftazidime	62.5	0.0	37.5	58.3	60.4	41.7	39.6	95.8	93.8	0.0	0.0	4.2	4.2	0.0	2.1
**CARBAPENEMS**															
Meropenem	100.0	0.0	0.0	100.0	100.0	0.0	0.0	100.0	100.0	0.0	0.0	0.0	0.0	0.0	0.0
**MONOBACTAMS**															
Aztreonam	58.3	4.2	37.5	81.3	81.3	18.7	18.8	68.8	68.8	4.2	4.2	4.2	4.2	22.9	22.9
**FLUOROQUINOLONES**															
Ciprofloxacin	72.9	4.2	22.9	75.0	72.9	25.0	27.1	79.2	81.3	2.1	2.1	10.4	10.4	8.3	6.3
**AMINOGLYCOSIDES**															
Amikacin	91.7	0.0	8.3	91.7	89.6	8.3	10.4	95.8	97.9	0.0	0.0	2.1	2.1	2.1	0.0
Tobramycin	75.0	0.0	25.0	77.1	77.1	22.9	22.9	93.8	93.8	0.0	0.0	2.1	2.1	4.2	4.2
**TETRACYCLINES**															
Tigecycline	93.8	0.0	6.3	100.0	100.0	0.0	0.0	93.8	93.8	0.0	0.0	0.0	0.0	6.3	6.3
**MISCELLANEOUS AGENTS**															
Colistin	93.8	0.0	6.3	100.0	100.0	0.0	0.0	93.8	93.8	0.0	0.0	0.0	0.0	6.3	6.3
Trimethoprim-Sulfamethoxazole	70.8	0.0	29.2	72.9	70.8	27.1	29.2	97.9	100.0	0.0	0.0	0.0	0.0	2.1	0.0

## Data Availability

All raw and processed sequencing data generated in this study are freely available in a GitHub repository accessible at https://github.com/NickVersmessen/Versmessen-et-al.-2024.-WGS-based-prokaryotic-typing, accessed on 16 August 2024.

## Supplementary Materials

The following supporting information can be downloaded at: https://www.mdpi.com/article/10.3390/diagnostics14161800/s1, SI 1. Protocol optimization for PromethION sequencing of up to 96 barcodes; SI 2. Schematic overview of the data-analysis workflow; SI 3. Gel view of the DNA analyzed by the Fragment AnalyzerTM System; SI 4. Protocol optimization and library sequencing features; SI 5. Read length histogram of the base called bases with outliers discarded; SI 6. Genome assembly quality parameters of six subdatasets with decreasing sizes for N = 9 isolates; SI 7. Pairwise analysis of WGS quality parameters between the complete and reduced datasets; SI 8. Sequence quality parameters for the complete and reduced datasets; SI 9. Similarity matrix depicting average nucleotide identity (ANI) and digital DNA–DNA hybridization (DDH) with MLST classifications; SI 10. Strain typing results for the complete and reduced datasets; SI 11. Pairwise analysis of the genome-based antibiotic resistance prediction and phenotypes between the complete (100%) and reduced (10%) datasets. References [65,66] were cited in Supplementary Materials.
