# Supplementary material for: Average Nucleotide Identity and Digital DNA-DNA Hybridization Analysis Following PromethION Nanopore-Based Whole Genome Sequencing Allows for Accurate Prokaryotic Typing
Source: Diagnostics (Basel). 2024 Aug 17;14(16):1800. doi: 10.3390/diagnostics14161800 (PMC11353866; doi:10.3390/diagnostics14161800)
Supplement: Supplementary file 1 [file diagnostics-14-01800-s001.zip › diagnostics-3061865-supplementary.pdf]

diagnostics

MDPI

Supplementary Materials

SI 1. Protocol optimization for PromethION sequencing of up to 96 barcodes.

We optimized and validated an existing library preparation protocol, originally designed for MinION and GridION and specifically tailored for sequencing up to 96 barcodes, for the PromethION platform [64]. Our approach was inherently shaped by the substantial capacity of the PromethION, ensuring that every modification considered was in line with maximizing its data output for each of the strains.

DNA repair and DNA tail preparation

The DNA repair and end-prep steps were executed by the protocol instructions, with one notable exception. To optimize our approach, we deviated from the protocol's recommendation of 400 ng bacterial DNA to commence with and employed the dilution rule to determine the initial starting amount of DNA. This adjustment was made to maximize DNA input for each isolate to retain more DNA in the final library. Considering the constraints of a maximum total volume of 12 µL per tube and the lowest measured DNA concentration with Qubit among the DNA extracts (which was 92.6 ng/µL), we could maximally include 1,110 ng of bacterial DNA per sample. Furthermore, we extended the incubation time during the preparation based on our experience with nanopore sequencing. Instead of the original 5 minutes, we prolonged incubation at 20 °C to 15 minutes in a thermal cycler.

Native barcode ligation (EXP-NBD196)

We doubled the original volume (0.75 µL) for the transfer of end-prepped DNA to retain more DNA after washing (**Supplemental Table S1**). Therefore, we also doubled the volume of native barcode solution (1.25 µL) to provide sufficient availability of barcode sequences in the solution for ligation. The end-prepped DNA was transferred with a focus on equimolar pooling as this greatly affects the sequencing coverage.

Supplemental Table S1. Composition of the reaction volumes during the native barcode ligation step using the EXP-NBD196 kit. The volume of each respective reagent is given for the original protocol and the optimized protocol.

Reagent	Original volume (µL)	Optimized volume (µL)
Nuclease-free water	3	1
End-prepped DNA (± 74 ng DNA/µL)	0.75	1.5
Native barcode	1.25	2.5
Blunt/TA Ligase Master Mix	5	5
EDTA 0.5 M pH 8	1	1
**Total**	**11**	**11**

The barcoded samples were combined into a 1.5 mL Eppendorf tube, resulting in a total mixture volume of 550 µL. We adjusted the volume ratio of Beckman Coulter™ Agencourt AMPure XP beads (Fisher Scientific) to the library pool from the initially suggested 0.4:1 to a 1:1 ratio to include more DNA. Thus, the mixture was then combined with an equal volume of 550 µL of beads. The incubation duration was extended to 30 minutes from the originally recommended 10 minutes at room temperature. Subsequent steps of the protocol were executed according to the provided instructions. Following completion, the DNA concentration of the eluted sample (35 µL) was quantified by Qubit, and the yield was calculated accordingly.

Adapter ligation and clean-up

The adapter ligation and clean-up procedure was executed according to the protocol with slight modifications [64]. The incubation times were extended from 10 to 15 minutes (reaction mix), 10 to 20 minutes (after addition beads), and 10 to 20 minutes (elution), based on our experience with nanopore sequencing.

To specifically enrich DNA fragments longer than 3 kbps while eliminating smaller fragments, we opted for the Long Fragment Buffer included in the kit. Additionally, we adjusted the beads-to-pool ratio to a 1:1 ratio instead of the initial 0.4:1. Consequently, 50 µL of beads were combined with the library, resulting in a total reaction mixture volume of 100 µL.

Finally, to retain a higher volume, the pellet was resuspended in 26 µL of elution buffer, an increase from the original 15 µL. Subsequently, the DNA concentration of the eluate (26 µL) was quantified using Qubit, and the yield, measured in ng of DNA recovered and recovery percentage (efficiency), was determined.

Priming and loading of the PromethION flow cell for sequencing

The subsequent preparation steps of the PromethION flow cell were executed according to the manufacturer's protocol for loading the library on the PromethION flow cell and sequencing [65]. We followed the manufacturer's guidelines for loading the flow cell with 5-50 fmol of the library, in this case equivalent to 7 µL of the final library pool (~ 50 fmol) to reach max advised capacity. To determine the fmol unit, we utilized the online 'dsDNA: Mass to/from Moles Converter' from NEBioCalculator (New England BioLabs Inc., Ipswich, USA) by using an average 20 kb DNA fragment length. Following the 72-hour sequencing period, the flow cell was optionally reloaded with another 7 µL from the pool to increase data output for explorative data analysis.

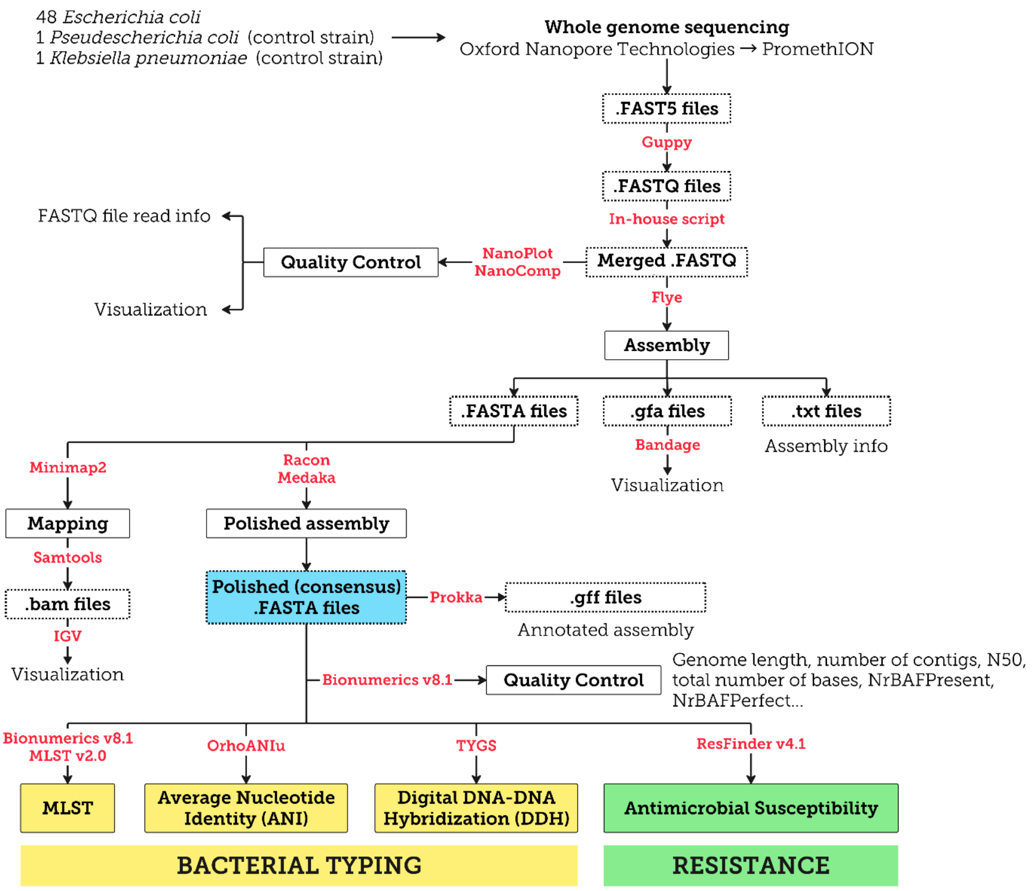

SI 2. Schematic overview of the data-analysis workflow. A selection of bacterial isolates is whole genome sequenced with the PromethION nanopore sequencing platform and the generated raw data files are processed as depicted. In this workflow, the output file types are depicted in dashed boxes while functional steps are shown in solid boxes. The software packages or scripts that were used to process the data are shown in red. The output data, containing the final FASTA files, used for subsequent analyses is highlighted in blue. MLST, multi-locus sequence typing; IGV, integrative genomics viewer; TYGS, type strain genome server.

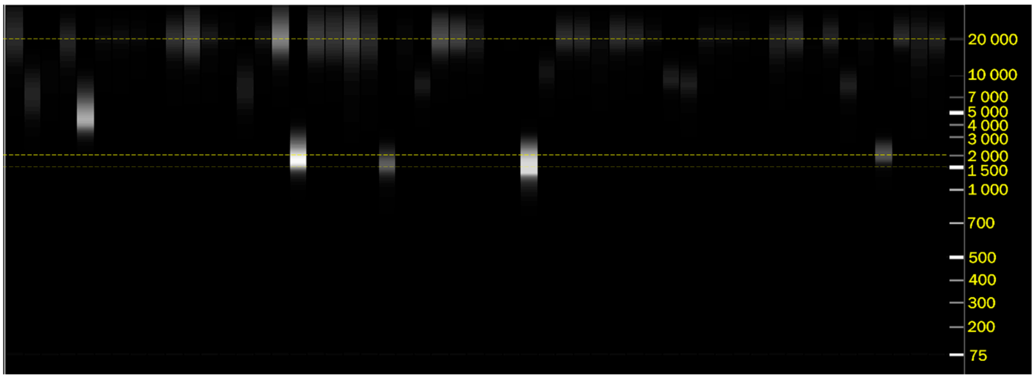

SI 3. Gel view of the DNA analyzed by the Fragment Analyzer™ System. The ladder (on the right) is shown in white markers with yellow numbers indicating the number of base pairs corresponding to each marker. The yellow dotted lines in the gel represent 20 kb (top) and 2 kb (bottom).

SI 4. Protocol optimization and library sequencing features

DNA yield

During an initial test library preparation, there was extremely poor DNA recovery after the washing steps, and it was not feasible to continue with this library preparation (data not included). A second and third library preparation was performed, the so-called pre-optimization preparation and the post-optimization preparation, respectively. Notably, since the initial test preparation failed, we had already increased (by 200%) the amount of DNA to commence with during the pre-optimization trial. The pre-optimization library preparation commenced with a total of 2,000 ng of DNA for the 50 isolates, amounting to 40 ng per well in the reaction mix of the native barcoding ligation part before the first washing step (as outlined in **Supplemental Table S2**). Notably, the original protocol recommended beginning with only half of this quantity, specifically 1,000 ng of DNA for the same set of 50 isolates, equating to 20 ng of DNA per well in the reaction mix of the native barcoding ligation part. However, we made this adjustment to ensure that a satisfactory amount of DNA remained available for loading onto the PromethION following the library preparation process. For the post-optimization library preparation, we started with 5,550 ng of DNA for the 50 isolates equal to 111 ng DNA per well in the reaction mix of the native barcode ligation part of the protocol (a total increase of 555% of the prescribed amount) (**Supplemental Table S2**).

Supplemental Table S2. DNA yield after each step protocol for the preparation of a library pool of 50 bacterial isolates. For the calculation of the molar units (in fmol), an average fragment length of 20 kb was used (cf. **SI 3**). A small volume was used each time between the protocol parts for DNA quantification and is included in the calculations.

Step of protocol	$DNA_{pre\text{-}wash}$ (ng) (fmol)	$DNA_{post\text{-}wash}$ (ng)(fmol)	DNA recovery (%)
Pre-optimization			
Native barcode ligation	2,000 (161.8)	353.5 (28.6)	**17.7**
Adapter ligation and cleanup	343.4 (27.8)	138.8 (11.2)	**40.4**
Total recovery	*2,000 ng (161.8 fmol)*	*138.8 ng (11.2 fmol)*	***6.9 %***
Post-optimization			
Native barcode ligation	5,550 (449.1)	4,120 (333.4)	**74.2**
Adapter ligation and cleanup	4,017 (325)	2,184 (176.7)	**54.4**
Total recovery	*5,550 ng (449.1 fmol)*	*2,184 ng (176.7 fmol)*	***39.4 %***

Combined, the increased start amount of DNA, the extended incubation times, and an adapted ratio of beads led to a yield increase in DNA recovery for the first part of the protocol (*native barcode ligation*) of 56.55%, and 13.94% for the second part (*adapter ligation and clean-up*) (**Supplemental Table S2**). The overall DNA recovery percentage pre- and post-optimization increased from 6.94% to 39.35% after optimization, a difference of 32.41%.

Library outcomes and flow cell loading

The final pool retained a total volume of 26 µL and contained approximately 2,184 ng DNA which is equivalent to 176.70 fmol. After quality control, a final pool of 22 µL (1,848 ng DNA or 149.50 fmol) remained. Subsequently, 7 µL of this pool was loaded onto the PromethION flow cell, equivalent to 588 ng DNA or 47.58 fmol, and again with 7 µL after a 72-hour sequencing period.

Sequenced read lengths

The read length histogram of the sequenced DNA fragments is shown in **SI 5**. The length of the median read (N50) was estimated to be 12.36 kb.

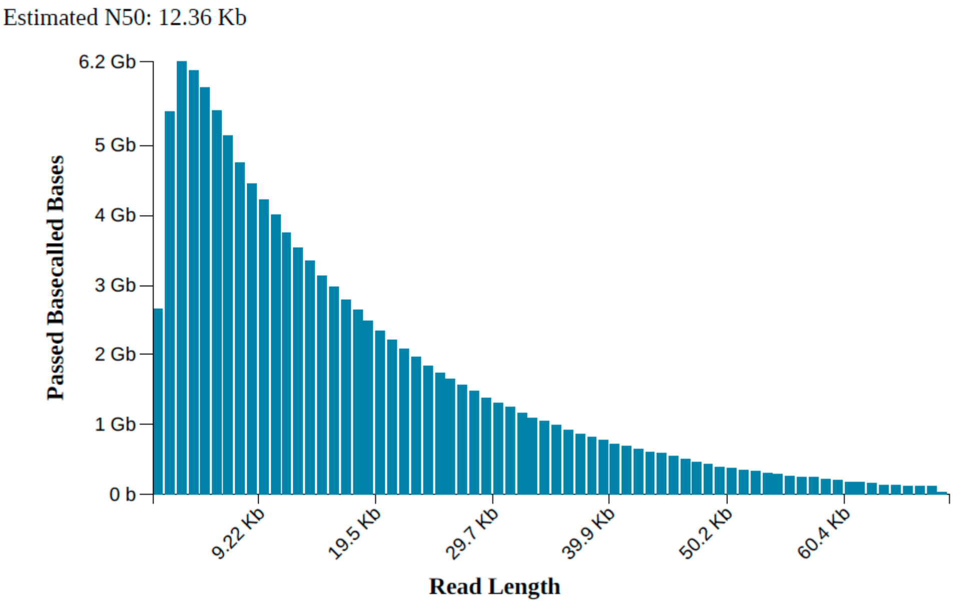

SI 5. Read length histogram of the base called bases with outliers discarded. Kb; kilobases, Gb, gigabases.

SI 6. Genome assembly quality parameters of six subdatasets with decreasing sizes for N = 9 isolates. *ref = similarity to the complete 100% (reference) data for the corresponding isolate; ANI, average nucleotide identity; bps, base pairs; DDH, DNA-DNA hybridization. Green rows indicate the smallest subdataset that delivers comparable quality data as the complete dataset, and red rows indicate the subdatasets where the quality of the data is decreased compared to the complete dataset. The color indication is calculated as follows: 75% subset ANI or DDH value – mean of ANI or DDH values from current and previous subdatasets = Diff. For ANI: (Diff > 0.02%) is considered significantly different, for DDH: (Diff > 0.1%) is considered significantly different. This empirical approach to investigate significant differences between the six subdatasets is indicative and was chosen based on the characteristics of this dataset. Emerging anomalies might indicate an issue with the WGS quality or with poorly chosen cut-off values for the methods investigated. In the dataset below, the above-described empirical approach could not be used to assess significant differences with the chosen parameter cut-off values for strain MTT033. Detailed investigation of the sequencing data for this strain however shows an outlier value of 0.70 for the WGS feature parameter NrBAFPerfect/NrBAFPresent (which is related to the total number of identified genes and was of poor quality) and is likely related to the cause.

Isolate ID	Subdataset (% reads)	Coverage (x)	Longest contig (bps)	Number of reads	ANI-to-ref* (%)	DDH-to-ref* (%)
**MTT004**	100	581	4,557,710	519,730	100	100
	75	434	4,557,715	389,257	99.89	99.70
	50	283	4,557,752	259,661	99.89	99.70
	25	147	4,557,698	130,563	99.88	99.70
	10	57	4,557,679	52,368	99.89	99.70
	5	28	4,557,478	26,098	99.89	99.70
	3	17	4,556,665	15,478	99.89	99.70
**MTT016**	100	424	3,764,663	405,857	100	100
	75	323	3,757,066	303,906	99.89	99.80
	50	215	3,756,815	202,747	99.89	99.80
	25	107	3,757,099	101,841	99.88	99.70
	10	41	3,757,015	40,840	99.89	99.70
	5	20	3,722,934	20,323	99.84	99.50
	3	11	2,296,113	12,027	99.75	99.50

**MTT026**	100	458	4,601,061	246,778	100	100
	75	341	4,601,066	184,883	99.89	99.70
	50	227	4,601,086	123,406	99.87	99.70
	25	114	4,601,116	61,948	99.89	99.70
	10	45	4,601,023	24,914	99.89	99.70
	5	22	4,600,742	12,389	99.89	99.70
	3	12	4,600,488	7,304	99.86	99.60
**MTT027**	100	221	4,780,347	182,637	100	100
	75	166	4,780,305	136,907	99.93	99.80
	50	110	4,780,358	91,250	99.93	99.80
	25	55	4,780,353	45,933	99.93	99.80
	10	22	4,780,958	18,465	99.91	99.60
	5	10	4,816,341	9,159	99.75	98.50
	3	6	1,093,366	5,403	99.02	90.90
**MTT033**	100	383	5,123,040	285,900	100	100
	75	286	5,123,068	214,099	99.94	99.80
	50	190	5,123,061	142,931	99.87	99.70
	25	95	5,122,977	71,655	99.90	99.70
	10	37	5,122,842	28,720	99.89	99.60
	5	19	5,122,361	14,330	99.84	99.40
	3	10	5,122,619	8,462	99.74	98.70
**MTT035**	100	301	5,159,262	272,708	100	100
	75	225	5,159,175	204,227	99.88	99.60
	50	150	5,159,200	136,365	99.89	99.70
	25	75	5,159,892	68,384	99.87	99.70
	10	29	5,159,635	27,451	99.86	99.70
	5	14	5,159,823	13,677	99.86	99.60
	3	9	761,976	8,086	99.72	97.10
**MTT037**	100	475	5,136,810	333,117	100	100
	75	353	5,136,815	249,589	99.98	100
	50	236	5,136,844	166,580	99.98	100
	25	118	5,136,772	83,697	99.97	100
	10	47	5,136,724	33,532	99.98	100
	5	23	5,136,389	16,695	99.91	99.90
	3	13	5,136,157	9,873	99.89	99.70
**MTT040**	100	484	5,097,250	327,111	100	100
	75	364	5,097,209	245,105	99.98	100
	50	241	5,097,206	163,589	99.94	100
	25	120	5,097,235	82,158	99.97	100
	10	49	5,097,175	32,910	99.94	100
	5	23	5,096,836	16,409	99.96	99.90
	3	14	5,096,629	9,703	99.89	99.70
**MTT050**	100	376	5,060,728	331,019	100	100
	75	281	5,060,742	248,029	99.96	100
	50	187	5,060,739	165,523	99.92	100
	25	93	5,060,740	83,160	99.99	100
	10	36	5,060,617	33,311	99.93	100
	5	17	5,060,294	16,593	99.92	99.90
	3	10	5,061,031	9,809	99.81	99.30

SI 7. Pairwise analysis of WGS quality parameters between the complete and reduced datasets. The polished FASTA files from each dataset were loaded in BioNumerics v.8.1 and WGS quality control analysis was performed. Two isolates, MTT001 and MTT031, were not included as these were included as control strains (indicated by blank values (NA)). bps, base pairs; N50, represents the length at which half of the assembled genome is contained in contigs or scaffolds of that size or larger.

	Complete dataset (100%)				Reduced dataset (10%)			
Isolate	Genome size (bps)	N50 (bps)	Contigs	Coverage	Genome size (bps)	N50 (bps)	Contigs	Coverage
MTT001	NA	NA	NA	411	NA	NA	NA	41
MTT002	5,266,861	5,133,524	6	370	5,205,964	5,133,290	3	36
MTT003	5,156,843	5,115,399	3	311	5,142,809	5,107,018	2	30
MTT004	4,558,973	4,558,973	1	582	4,596,467	4,558,989	2	57
MTT005	4,573,156	4,558,984	2	260	4,564,212	4,559,325	2	25
MTT006	4,561,061	4,558,223	5	413	4,570,397	4,559,452	3	40
MTT007	4,627,907	4,627,907	1	373	4,628,573	4,628,573	1	37
MTT008	4,563,174	4,558,559	5	311	4,572,109	4,558,633	5	30
MTT009	4,627,910	4,627,910	1	565	4,627,917	4,627,917	1	56
MTT010	4,643,160	4,531,181	2	338	4,624,692	4,512,600	2	33
MTT011	4,640,314	4,506,743	3	361	4,641,300	4,496,762	3	35
MTT012	5,162,203	5,151,434	3	409	5,163,837	5,153,428	3	41
MTT013	5,504,247	5,116,375	4	360	5,484,979	5,115,455	5	35
MTT014	5,401,150	3,782,318	3	364	5,296,474	3,745,800	3	35
MTT015	5,379,998	3,805,343	3	354	5,269,848	3,722,157	3	35
MTT016	5,417,878	3,765,603	3	424	5,300,234	3,758,389	3	41
MTT017	5,324,544	3,762,310	3	318	5,321,182	3,758,174	3	31
MTT018	5,166,957	4,897,489	8	427	5,179,325	4,897,422	8	41
MTT019	5,158,691	4,895,714	8	394	5,158,068	4,895,074	7	39
MTT020	5,213,781	4,894,162	10	464	5,166,909	4,893,245	8	46
MTT021	5,168,511	2,679,837	14	184	5,110,786	259,382	58	18
MTT022	5,298,062	5,050,262	4	450	5,293,559	5,050,333	4	45
MTT023	6,268,206	772,895	52	271	6,022,166	871,130	48	28
MTT024	5,477,511	5,096,719	9	374	5,569,072	5,082,239	14	36
MTT025	4,776,502	4,602,435	2	412	4,776,499	4,602,417	2	41
MTT026	4,776,480	4,602,406	2	458	4,776,540	4,602,458	2	45
MTT027	5,201,121	4,782,306	5	221	5,129,631	4,782,833	4	22
MTT028	5,802,419	5,385,327	15	225	5,806,171	5,385,990	16	22
MTT029	5,263,992	5,105,543	2	291	5,264,256	5,105,748	3	28
MTT030	4,805,810	4,602,407	8	502	4,809,248	4,602,265	10	50
MTT031	NA	NA	NA	511	NA	NA	NA	51
MTT032	5,384,597	5,034,384	6	282	5,323,365	5,033,645	5	28
MTT033	5,450,954	5,126,175	7	383	5,437,296	5,125,293	7	37
MTT034	5,427,961	5,126,111	5	500	5,425,751	5,125,493	6	49
MTT035	5,161,076	5,161,076	1	301	5,161,723	5,161,723	1	29
MTT036	5,406,768	5,137,748	4	404	5,338,751	5,138,382	3	39
MTT037	5,338,973	5,138,586	3	475	5,363,534	5,138,519	3	47
MTT038	5,231,812	5,146,269	2	563	5,146,188	5,146,188	1	56
MTT039	5,146,226	5,146,226	1	567	5,146,164	5,146,164	1	56
MTT040	5,229,992	5,098,622	2	484	5,234,672	5,098,972	3	49
MTT041	5,320,215	5,098,709	5	459	5,319,654	5,098,360	4	46
MTT042	4,971,317	4,971,317	1	564	5,122,780	4,970,642	5	56
MTT043	5,059,009	4,904,925	6	512	5,060,645	4,904,137	7	51
MTT044	5,074,229	4,782,384	5	550	5,099,726	4,782,359	6	52
MTT045	5,252,988	5,067,539	3	443	5,252,946	5,067,545	3	44
MTT046	5,113,163	4,894,212	2	452	5,056,320	4,894,224	2	44
MTT047	5,422,342	5,164,204	4	447	5,428,950	5,164,267	4	44
MTT048	5,424,558	5,166,415	5	384	5,427,256	5,165,867	6	38
MTT049	5,283,196	5,024,820	7	374	5,259,553	5,024,941	6	37
MTT050	5,339,425	5,062,291	5	376	5,296,681	5,062,329	5	36

SI 8. Sequence quality parameters for the complete and reduced datasets. bps, base pairs; Q1, first quantile; Q3, third quantile; NrBAFPresent, all assembly-based calls, including perfect (100%) matches and non-perfect matches; NrBAFPerfect, all assembly-based calls that have a perfect match with an allele in the allele database; N50, the length at which half of the assembled genome is contained in contigs or scaffolds of that size or larger.

		Length (bps)	Contigs	Number of bases ACGT (bps)	N50 (bps)	NrBAFPresent	NrBAFPerfect	NrBAFPerfect/NrBAFPresent	Coverage (x)
Complete dataset (100%)	**Min.**	4,558,973	1	4,558,973	772,895	3,873	2,632	0.6393	184.0
	**Q1**	5,037,086	2	5,037,086	4,591,550	4,113	3,656	0.8633	355.5
	**Median**	5,221,886	4	5,221,886	4,938,121	4,232	4,024	0.9706	406.5
	**Mean**	5,163,046	5.438	5,163,046	4,703,756	4,199	3,776	0.9006	404.6
	**Q3**	5,381,148	6	5,381,148	5,118,809	4,296	4,131	0.9774	462.8
	**Max.**	6,268,206	52	6,268,206	5,385,327	4,723	4,415	0.9847	582.0
Reduced dataset (10%)	**Min.**	4,564,212	1	4,564,212	259,382	3,878	2,619	0.6231	18.0
	**Q1**	5,059,564	2.750	5,059,564	4,591,562	4,160	3,388	0.8043	35.0
	**Median**	5,173,117	3	5,173,117	4,937,390	4,224	3,829	0.9272	39.5
	**Mean**	5,145,316	6.375	5,145,316	4,651,574	4,199	3,619	0.8635	39.8
	**Q3**	5,320,036	6	5,320,036	5,117,914	4,297	3,942	0.9400	46.0
	**Max.**	6,022,166	58	6,022,166	5,385,990	4,625	4,110	0.9670	57.0

0

diagnostics

Isolate name	Strain designation	MLST	MLST color
**MTT005**	BL21+Lys	93	
**MTT008**	BL21+KJE7	93	
**MTT004**	BL21	93	
**MTT006**	BL21+G-KJE8	93	
**MTT007**	BL21Gro7	6142	
**MTT010**	Top10	1060	
**MTT011**	Top10+P	1060	
**MTT026**	CC3B	10	
**MTT030**	DC1	10	
**MTT009**	BL21+G-Tf2	6142	
**MTT025**	CC3A	10	
**MTT027**	CC4A	167	
**MTT044**	BM1	167	
**MTT021**	CM4	10	
**MTT019**	CM2	10	
**MTT018**	CM1	10	
**MTT020**	CM3	10	
**MTT023**	CC2A	88	
**MTT024**	CC2B	88	
**MTT022**	CC1A	88	
**MTT029**	CC1B	88	
**MTT047**	BA1	88	
**MTT050**	BY2	88	
**MTT048**	BA2	88	
**MTT049**	BY1	88	
**MTT046**	BM3	58	
**MTT034**	DC5	783	
**MTT032**	DC3	783	
**MTT033**	DC4	783	
**MTT028**	CC4B	2141	
**MTT012**	Congo1	131	
**MTT036**	DS1	12	
**MTT037**	DS2	12	
**MTT038**	SH2D0	12	
**MTT039**	SH2D3	12	
**MTT013**	Congo2	12	
**MTT042**	SH11D0	12	
**MTT043**	SH11D3	12	
**MTT016**	BC3	73	
**MTT017**	BC4	73	
**MTT014**	BC1	73	
**MTT015**	BC2	73	
**MTT002**	ATCC2	73	
**MTT035**	DS-Sister	73	
**MTT003**	ATCC3	127	
**MTT045**	BM2	95	
**MTT040**	SH8D0	390	
**MTT041**	SH8D3	390	

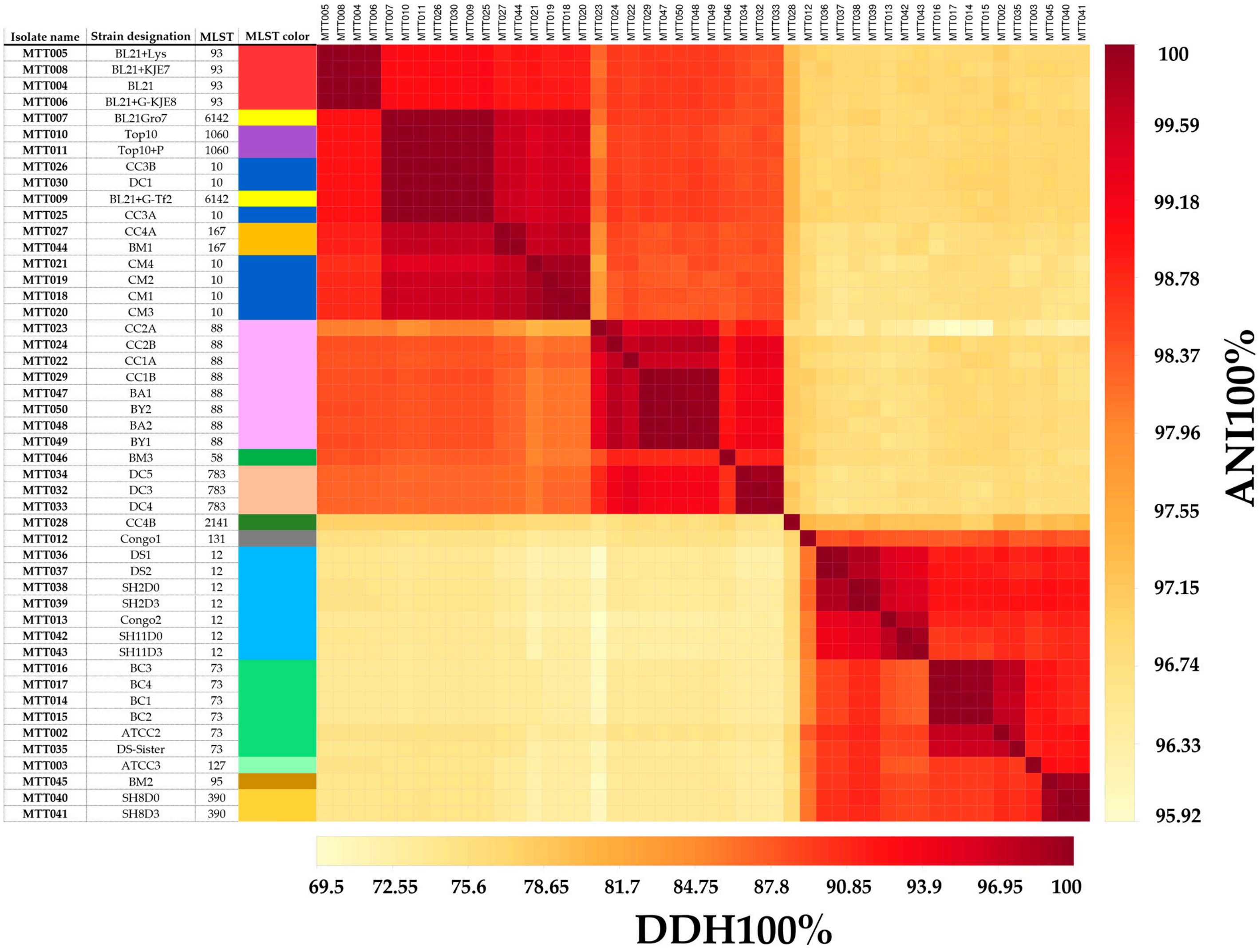

SI 9. **Similarity matrix depicting average nucleotide identity (ANI) and digital DNA–DNA hybridization (DDH) with MLST classifications.** The matrix illustrates ANI and DDH values with the bottom left section representing the DDH values and the upper right section displaying ANI values for the complete dataset. The analysis exclusively includes *E. coli* isolates. On the left side, strain IDs (first two columns) are presented alongside their MLST classifications (third column), with each unique class color-coded for distinction. Visualization was performed using the MAniR tool.

SI 10. Strain typing results for the complete and reduced datasets. MTT001 and MTT031 were not analyzed as these isolates are not *E. coli*. * : no decisive classification could be found; the class corresponds to the closest match in the reference database. – : MLST class was not found. CGE, Center for Genomic Epidemiology; BN, BioNumerics; (wg)MLST, (whole genome) multi-locus sequence type. Concordances and discordances between the two datasets, for the same tool, are marked in green and red, respectively. For both datasets, discordances between the CGE and the BN tool are shown in white font.

	Complete dataset (100%)		Reduced dataset (10%)	
**Isolate**	**CGE MLST**	**BN wgMLST**	**CGE MLST**	**BN wgMLST**
MTT002	ST73	ST73	ST73	ST73
MTT003	ST127*	–	ST127	ST127
MTT004	ST93	ST93	ST93	ST93
MTT005	ST93	ST93	–	–
MTT006	ST93	ST93	ST93	ST93
MTT007	ST6142	ST6142	ST6142	ST6142
MTT008	ST93	ST93	ST93	ST93
MTT009	ST6142	ST6142	ST6142	ST6142
MTT010	ST1060	ST1060	ST1060	ST1060
MTT011	ST1060	ST1060	ST1060	ST1060
MTT012	ST131	ST131	ST131*	–
MTT013	ST12*	–	ST12*	–
MTT014	ST73	ST73	ST73	ST73
MTT015	ST73	ST73	ST73	ST73
MTT016	ST73	ST73	ST73	ST73
MTT017	ST73	ST73	–	–
MTT018	ST10	ST10	ST10	ST10
MTT019	ST10	ST10	ST10	ST10
MTT020	ST10	ST10	ST10	ST10
MTT021	ST10	ST10	ST10*	–
MTT022	ST88	ST88	ST88	ST88
MTT023	ST88	ST88	–	–
MTT024	ST88	ST88	ST88*	–
MTT025	ST10	ST10	ST10	ST10
MTT026	ST10	ST10	ST10	ST10
MTT027	ST167	ST167	ST167	ST167
MTT028	ST2141	ST2141	ST2141	ST2141
MTT029	ST88	ST88	ST88	ST88
MTT030	ST10	ST10	ST10*	–
MTT032	ST783	ST783	ST783	ST783
MTT033	ST783	ST783	ST783	ST783
MTT034	ST783	ST783	ST783	ST783
MTT035	ST73	ST73	ST73	ST73
MTT036	ST12	ST12	ST12	ST12
MTT037	ST12	ST12	ST12	ST12
MTT038	ST12	ST12	ST12	ST12
MTT039	ST12	ST12	ST12	ST12
MTT040	ST390	ST390	ST390	ST390
MTT041	ST390	ST390	ST390	ST390
MTT042	–	–	ST12*	–
MTT043	–	–	ST12*	–
MTT044	ST167	ST167	ST167	ST167
MTT045	ST95	ST95	ST95	ST95
MTT046	ST58	ST58	ST58	ST58
MTT047	ST88	ST88	ST88	ST88
MTT048	ST88	ST88	ST88	ST88
MTT049	ST88*	–	ST88*	–
MTT050	ST88	ST88	ST88	ST88
**MLST result (including closest matches) %**	95.8%	89.6%	93.8%	77.1%

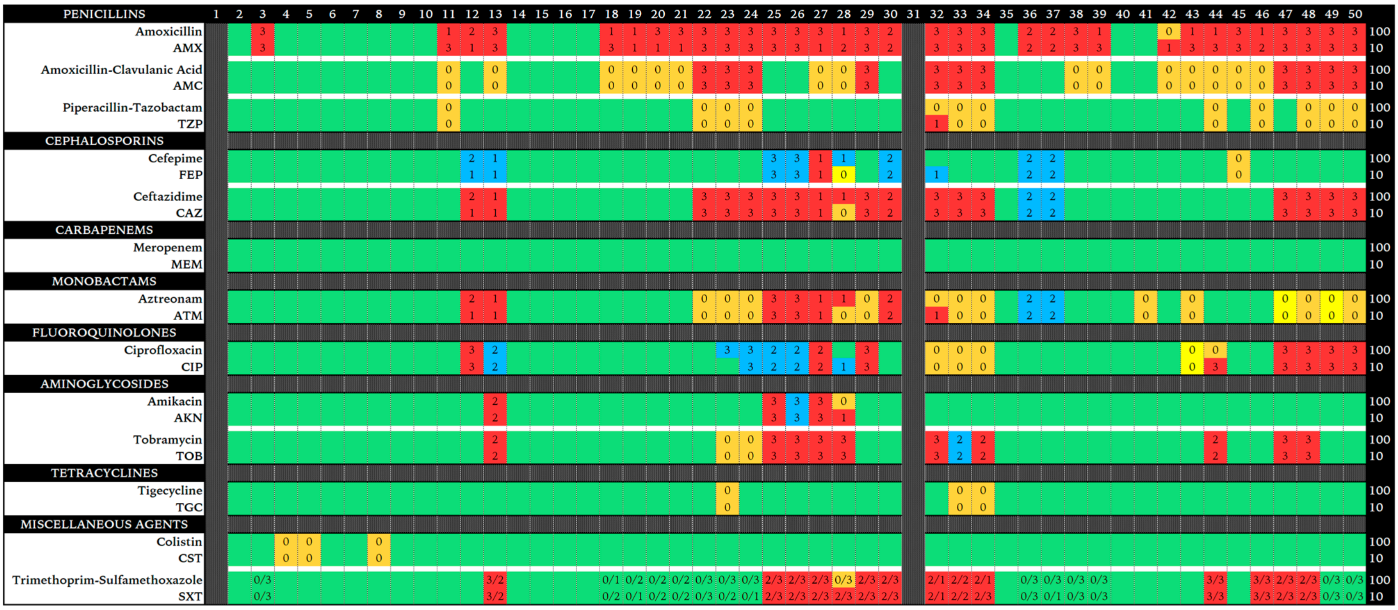

SI 11. Pairwise analysis of the genome-based antibiotic resistance prediction and phenotypes between the complete (100%) and reduced (10%) datasets. The figure presents an overlay of WGS prediction-based antibiotic resistance using the ResFinder tool and resistance phenotypes (MIC assay). Green (susceptible) and red (resistant) both indicate concordance between genome-based prediction and the resistance phenotype. The remaining colors indicate discrepancies where yellow indicates a minor error as intermediate/susceptible (MIC/WGS prediction) observation, blue is a major error as susceptible/resistant (MIC/WGS) observation, and orange a very major error as resistant/susceptible (MIC/WGS) observation. Isolates are numbered from 1 to 50. Isolates 1 and 31 were not analyzed (non-E. *coli* strains). The dataset is specified on the right (100% or 10%). The numbers in the different cells indicate the score classification of resistance by ResFinder. For the combination antibiotics trimethoprim-sulfamethoxazole, there was no score classification by ResFinder for the two combined and only for the two antibiotics separately. Therefore, the score is shown as trimethoprim score/sulfamethoxazole score. If at least one of the two antibiotics trimethoprim or sulfamethoxazole was scored as susceptible, the overall phenotype was set to susceptible.
